# Pasteurella multocida Spondylodiscitis in an Immunocompetent Patient

**DOI:** 10.7759/cureus.78602

**Published:** 2025-02-06

**Authors:** Débora A Alves, João Trêpa, Isabel Ramos, Cristina Valente

**Affiliations:** 1 Infectious Diseases Department, Centro Hospitalar e Universitário de Coimbra, Unidade Local de Saúde de Coimbra, Coimbra, PRT; 2 Intensive Care Unit, Unidade Local de Saúde de Viseu Dão-Lafões, Viseu, PRT

**Keywords:** cervical spine infection, immunocompetent patient, pasteurella multocida, septic shock, zoonosis

## Abstract

We present the case of an 82-year-old woman, previously independent in activities of daily living, who developed fever, myalgias, and headache over one week. Two weeks earlier, she had been treated with antibiotics for a lower respiratory tract infection. The patient had no history of immunosuppression and was a pet owner. She was admitted to the emergency department (ED) with a fever and multiple perforating wounds on her hands. Laboratory findings revealed elevated inflammatory markers, including C-reactive protein and procalcitonin, without an obvious infectious source. During observation and further investigations in the ED, her clinical condition rapidly deteriorated, requiring vasopressor support and subsequent transfer to the intensive care unit (ICU). Blood and urine cultures were obtained, and empirical broad-spectrum antibiotics were initiated. In the ICU, the patient developed severe neck pain with functional limitations. Cervical magnetic resonance imaging (MRI) revealed spondylodiscitis with C3-C4-C5 paramedian epidural empyema. Blood cultures identified *Pasteurella multocida*, and the patient was treated with intravenous ceftriaxone (2g every 12 hours) for eight weeks. A follow-up MRI at the end of treatment showed significant improvement, with a marked reduction in empyema and no need for surgical intervention. The patient was managed with a cervical collar and physical rehabilitation. At discharge, she had made substantial functional recovery, with no neurological deficits, and her inflammatory markers had returned to baseline.

## Introduction

*Pasteurella multocida* is a gram-negative, non-motile, facultative anaerobic, penicillin-sensitive coccobacillus. It belongs to the Pasteurellaceae family and is a common commensal organism in the upper respiratory tract of many animals, particularly cats and dogs. While generally harmless to its animal hosts,* P. multocida* can cause zoonotic infections in humans, typically following animal bites, scratches, or licks, especially to broken skin [1].

The carrier rate of *P. multocida* is 70%-90% in cats and 20%-50% in dogs. Cat bites pose a higher risk of infection due to the greater colonization of* P. multocida* in cats and the fact that their small, sharp teeth cause deeper wounds [2,3]. *P. multocida* is the most common pathogen isolated from wounds following animal bites or scratches. However, other microorganisms, such as *Bartonella henselae*, *Clostridium tetani*, and *Staphylococcus aureus*, should also be considered in the differential diagnosis [4].

*P. multocida* infections present with a wide range of clinical manifestations, including skin and soft tissue infections, bacteremia, respiratory tract infections (e.g., pneumonia, epiglottitis), endocarditis, intra-abdominal infections and, albeit rarely, spinal infections such as spondylodiscitis and spinal epidural abscesses, the latter being, although uncommon, particularly in immunocompetent individuals, can be a life-threatening infection [5-8]. Nevertheless, clinicians should maintain a high index of suspicion for this pathogen when evaluating patients with spinal infections, as prompt diagnosis and treatment are essential for a favorable outcome. The severity of infection can vary depending on various factors, including the site of infection, the immune status of the individual, and the specific strain of *P. multocida* involved.

Spondylodiscitis is a rare but potentially life-threatening infection involving the intervertebral disc and adjacent vertebral bodies. It can result from bacterial, fungal, or mycobacterial infections, and its management requires early identification and appropriate antimicrobial therapy to prevent severe complications. This case report describes a septic shock due to *P. multocida* bacteremia and subsequent bone and spinal infection in an elderly patient. The patient showed clinical resolution following medical therapy, underscoring the potential for successful outcomes in such cases, even in the absence of known immunosuppression.

## Case presentation

**Figure 1:**
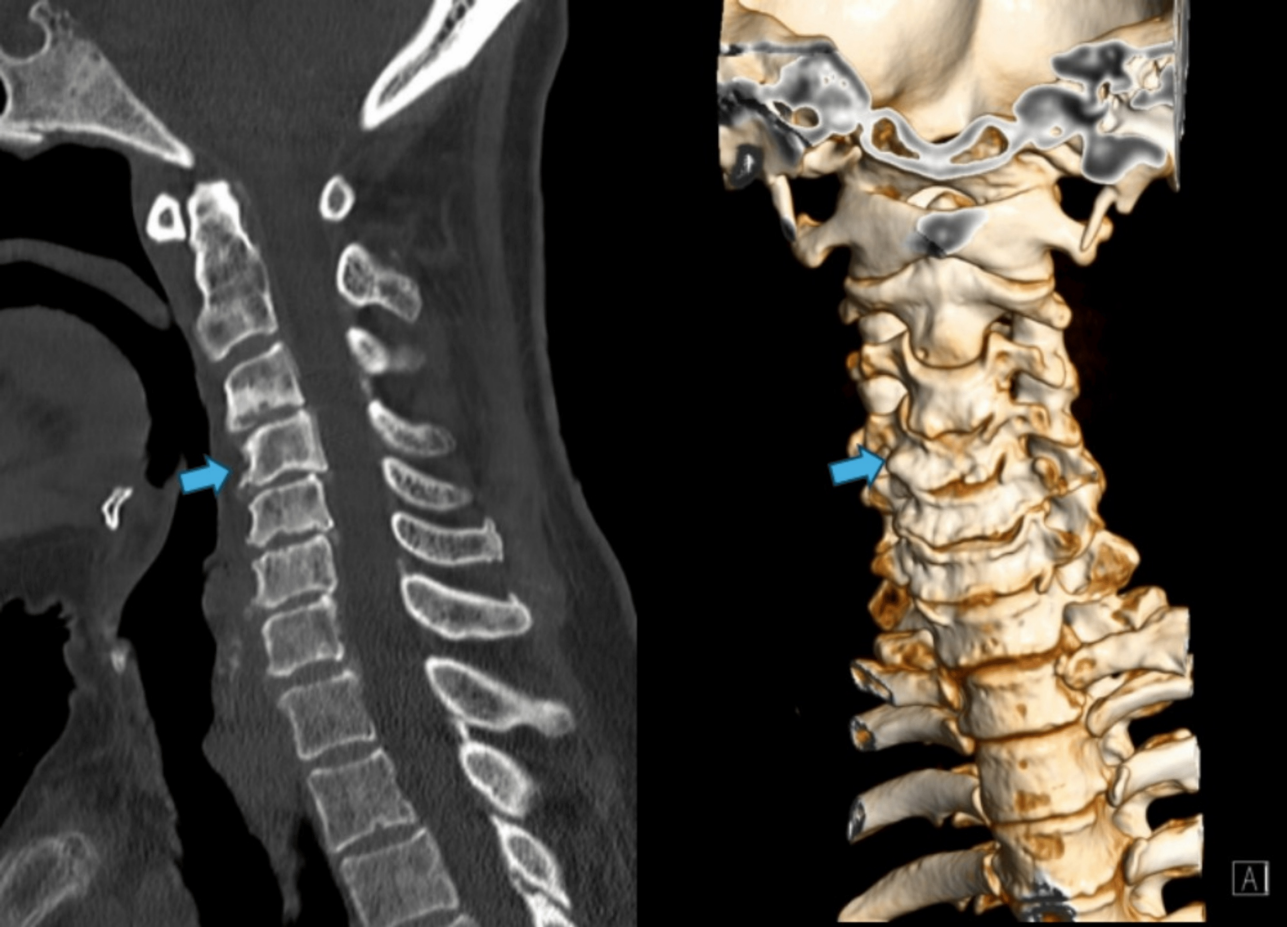
Sagittal and coronal plane on CT imaging (June 2022) There are no traces of recent fractures in the discovered planes, nor images suggestive of intracanal hematomas. The spinal canal has specific dimensions, but is narrow due to degenerative changes, particularly in the segment between C3 and C6. Kyphotic inversion of the cervical curvature, with incipient retrolisthesis of C4, and slight scoliotic deviation of right convexity, centered on C3-C4, in the study position. The spinal canal has specific dimensions, although it is narrow due to degenerative changes, particularly in the segment between C3 and C6 (arrows), highlighting discopathies with reduced interbody height and sclerosis of the vertebral platforms, uncarthroses, disc protrusions/disc-osteophytes, mild interapophyseal arthrosis, and calcifications of the yellow ligaments.

**Figure 2:**
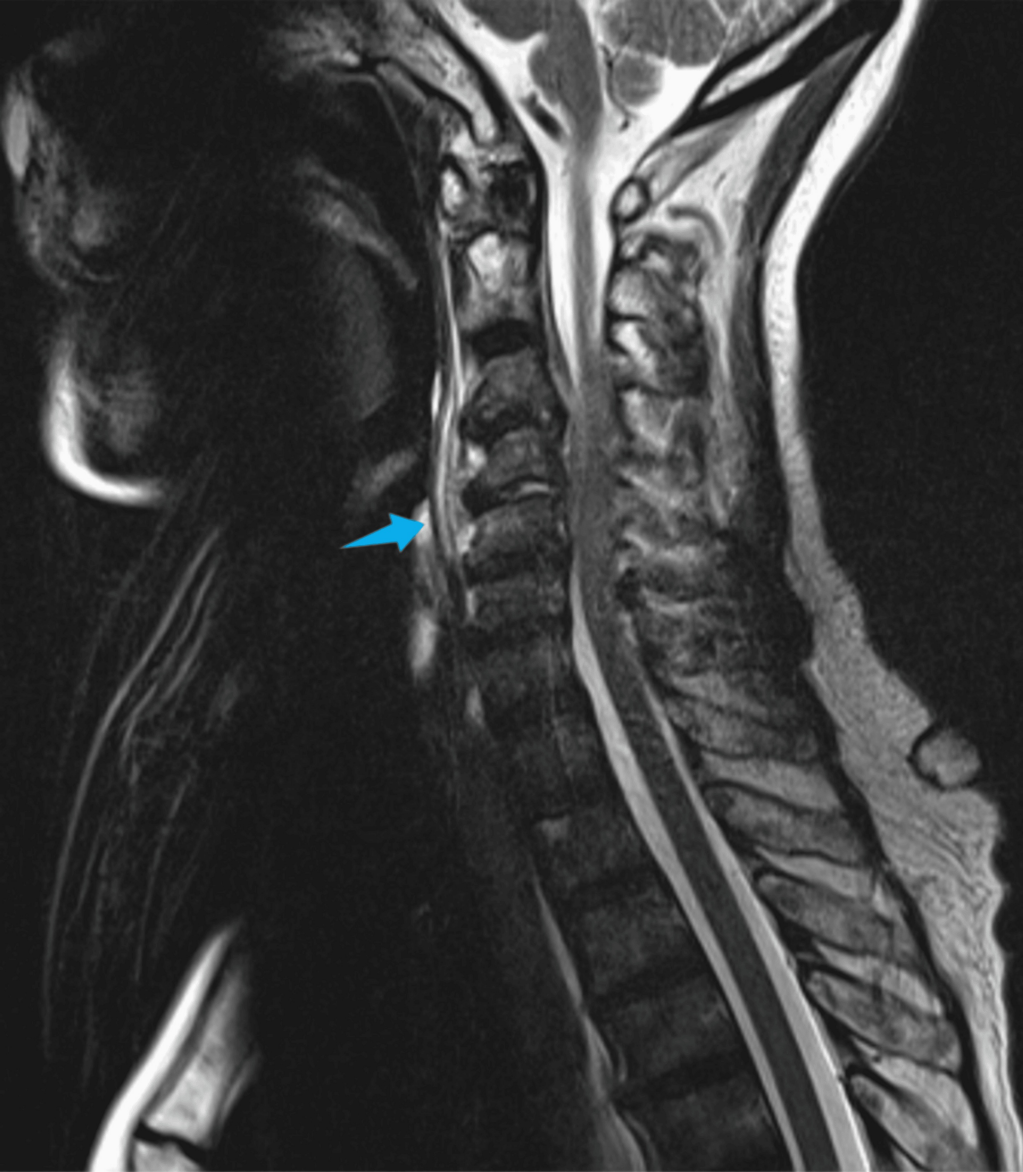
Sagittal T2-weighted MRI (June 2022) Inflammatory changes were identified in the C4-C5 intervertebral disc, as well as an anterior epidural collection with left paramedian predominance, between the body of C2 and the lower somatic platform of C5. This finding is consistent with the diagnosis of empyema (arrow). The collection extends through the foramina to the para and pre-vertebral space adjacent to the bodies of C3, C4, and C5.

**Figure 3:**
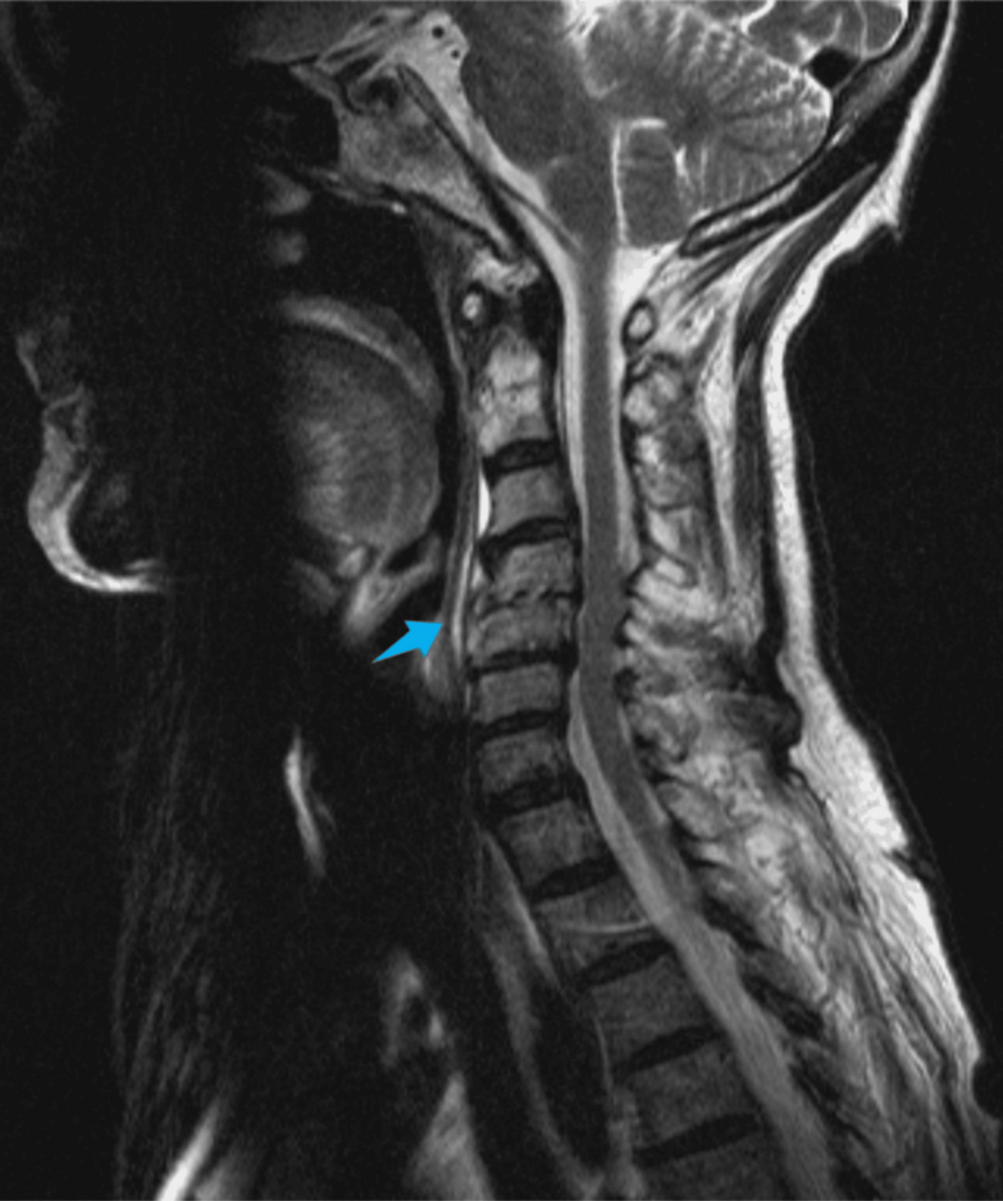
Sagittal T2-weighted MRI (August 2022) Compared to the previous MRI performed in June, this follow-up imaging demonstrates global improvement. The findings show a reduction in the height of the C4-C5 intervertebral disc and slight irregularities of the vertebral platforms with discreet signal reinforcement of these and the disc after contrast (arrow); marked reduction of the previously existing epidural component, currently only the presence of discrete signal reinforcement after contrast at the epidural level is identified, extending approximately from the plane of C3 to the plane of the lower vertebral platform of C5; additionally, there is a reduction of the peri-vertebral component.

An 82-year-old female patient (Katz score of 6, i.e., independent patient) presented to the emergency department (ED) with a one-week history of fever, myalgias, and headache. The patient had received empiric antibiotic therapy (amoxicillin and clavulanic acid) 15 days prior for suspected lower respiratory tract infection. The patient's medical history included controlled hypertension, dyslipidemia, and atrial fibrillation. The patient also had a history of owning 19 cats and two dogs. On physical examination, the patient had an axillary temperature of 38 degrees Celsius, and skin lesions suggestive of an animal (cat) bite were detected on both hands, without inflammatory signs. Body mass index low weight: BMI ≤ 18.5 kg/m^2^. The remaining physical examination was unremarkable. Initial laboratory studies revealed an elevated white blood cell count (11.100 per microliter), with neutrophil predominance (90%), and a very significant increase in C-reactive protein (44.98 mg/dL, reference < 0.50) and procalcitonin (82 ng/mL, reference 0-0.5). Table 1 shows the patient's initial analysis and her evolution during hospitalization. Serum albumin and total protein values ​​were normal. The patient’s clinical condition rapidly worsened, developing hypotension, fever, and oligoanuria, needing vasopressor support. Despite the absence of a specific infectious focus, septic shock was suspected. Blood and urine cultures were obtained, and empirical antibiotic therapy with piperacillin and tazobactam was initiated. The patient was admitted to the intensive care unit (ICU) with a Glasgow Coma Score of 13, where she developed persistent neck pain and limited mobility within the first 24 hours. Piperacillin/tazobactam was switched to meropenem in the ICU. A cervical computed tomography (CT) scan revealed cervical spondylarthrosis and dyscarthrosis with associated root compression, but no signs of infection (Figure 1). Given the high suspicion of an infectious process, magnetic resonance imaging (MRI) was performed, revealing C4-C5 spondylodiscitis with paramedian epidural empyema, extending into the para/pre-vertebral space adjacent to C3-C4-C5 across the foramina (Figure 2). Blood cultures were positive for *P. multocida. *Cultures for mycobacteria and fungus were negative and antibodies for *Coxiella,* *Brucella*, and HIV were negative. The final diagnosis was C4-C5 cervical spondylodiscitis secondary to *P. multocida *bacteremia, complicated by epidural empyema, which was likely associated with the patient’s risk factors related to animal exposure. After discussion with a multidisciplinary team, including radiology and neurosurgery specialists, and due to the lesion’s location and the risks associated with surgery, it was decided to pursue medical management alone. The patient was switched to intravenous ceftriaxone 2g every 12 hours for eight weeks, due to its safer profile in renal impairment, since the creatinine clearance was less than 30 mL/min. A trans-thoracic echocardiogram was performed, and no valvular vegetations were observed. The patient showed continued improvement, as demonstrated by follow-up imaging (Figure 3) after two months of antibiotic therapy and there was no need for intubation. She was discharged asymptomatic. Six months post-discharge, the patient remained stable from a clinical and radiological point of view, with no recurrence of the infection.

**Table 1:** Patient´s analytical evolution during hospitalization MCV - mean corpuscular volume; INR - international normalized ratio; PT - prothrombin time; APTT - partial thromboplastin time; LDH - lactate dehydrogenase; AST - aspartate transferase; ALT - alanine transaminase; GGT - gamma-glutamyl transferase; CK - creatine kinase; PCT - procalcitonin.

Day of admission		Hospital admission					Hospital discharge
Blood tests	Reference values	Day 0	Day 1	Day 3	Day 10	Day 51	Day 81
Hemoglobin (g/dL)	13.5-17.5	11.3	13.8	10.8	11.1	7.5	8.2
MCV (fL)	80-100	83.1	81.8	79.1	84.9	88.2	90.7
Leukocytes (x10^9/L)	3.90-10.2	4.8	11.1	17.3	9.9	2.1	2.7
Lymphocytes (x10^9/L)	1.10-4.50	0.44	0.48	1.03	0.64	0.57	0.72
Neutrophils (x10^9/L)	1.50-7.70 (38% - 75%)	4.15 (86%)	10.03 (90%)	15.33 (89%)	8.75 (88%)	0.86 (41%)	1.35 (50%)
Platelets (x10^9/L)	150-450	84	61	81	210	181	142
INR	0.8 - 1.2	1.95	1.54	1.15	1.04	0.91	0.96
PT (sec)	9.4 - 12.5	11.6	18.1	13.4	12.1	10.5	11.1
APTT (sec)	23.4 - 35.4	32.2	32.9	28.3	24.2	33.9	29.0
Creatinine (mg/dL)	0.72 - 1.18	1.33	2.93	4.01	1.1	0.97	1.53
LDH (U/L)	< 248	291	313	381	318	274	242
AST (U/L)	< 35	45	53	57	42	15	20
ALT (U/L)	<45	18	20	19	10	7	11
Alkaline phosphatase (U/L)	30 - 120	69	58	151	153	91	89
GGT (U/L)	< 55	58	46	71	115	44	64
Total bilirubin (mg/dL)	0.2 - 1.2	1	1	0.6	0.7	0.2	0.4
CK (U/L)	< 171	937	281	116	14	15	16
C-reactive protein (mg/dL)	< 0.50	22.63	44.98	10.62	16.39	3.03	0.52
PCT (ng/mL)	0 - 0.5	14.5	82	27.6	0.88	0.57	-

## Discussion

Infections caused by *P. multocida* are well-established zoonotic diseases, typically transmitted through animal bites or scratches. The clinical presentation of* P. multocida* infections is highly variable and depends on factors such as the site of infection and the host’s underlying health status. Localized infections, including cellulitis, abscesses, and lymphadenitis, are the most common manifestations and typically arise at the site of inoculation following an animal bite or scratch. However, *P. multocida *infections should be considered even in cases where the infection site is distant from the point of exposure, scratch, or lick, particularly in high-risk individuals [1]. Systemic infections, including bacteremia, pneumonia, and meningitis, though less common, can be severe and potentially life-threatening, especially in individuals with pre-existing comorbidities [9]. Several factors can increase the susceptibility to severe *P. multocida i*nfections. Immunocompromised individuals, such as those with HIV/AIDS or undergoing chemotherapy, are at heightened risk for more severe outcomes. Other underlying medical conditions, including diabetes mellitus, chronic liver disease, and chronic respiratory diseases, further predispose patients to complicated infections [2,9]. Additionally, both very young and elderly individuals are particularly vulnerable to adverse outcomes due to potentially weakened immune systems, which increases the risk of severe infections. Delayed initiation of appropriate treatment can exacerbate infections and worsen prognosis.

The diagnosis of* P. multocida* infection relies on a combination of clinical presentation, epidemiological history, and laboratory testing. The gold standard is culture of the infected site, complemented by Gram staining and biochemical tests for organism identification. In some cases, imaging studies, such as x-rays or CT scans, may be required to assess the extent of infection, especially in cases involving osteomyelitis or deep tissue involvement. In the described clinical case, the epidemiological history (exposure to multiple cats) was significant. Despite a Clinical Frailty Scale score of 3 (managing well), advanced age and low body weight remained risk factors for the infection.

*P. multocida* is generally susceptible to various antibiotics, including penicillin, amoxicillin-clavulanate, third-generation cephalosporins, and fluoroquinolones [4]. The selection of an appropriate antibiotic and treatment duration is determined by the severity and localization of the infection, as well as the patient’s medical history and any potential allergies [4]. Oral antibiotics are typically sufficient for localized infections; however, intravenous therapy may be required for more severe or systemic infections. Though susceptible to all tested antimicrobials, it presented a challenge due to both acute kidney injury and complex involvement of bone and epidural tissue. Using ceftriaxone is a sensible choice given its pharmacokinetic profile, which does not require dose adjustments for renal impairment. The decision to aim for a six-week course of treatment aligns with most experts and guidelines for bone and epidural infections, where prolonged therapy is necessary to achieve adequate bone penetration and prevent relapse [10].

The risk associated with surgery was primarily due to the location of the lesions in the cervical region. However, since there was no evidence of neurological impairment, a decision was made to pursue only medical treatment. Prevention of *P. multocida* infections involves prompt cleaning and disinfection of any animal bites or scratches. Individuals at high risk for severe infections should also seek immediate medical attention if exposed to animals or when experiencing symptoms suggestive of infection.

## Conclusions

This case report emphasizes the importance of considering *P. multocida* as a potential causative agent of spinal infections, even in the absence of a clear history of animal bites or exposure. *P. multocida* should be particularly suspected in elderly patients with chronic diseases who have frequent contact with domestic animals, especially cats and dogs. To prevent pasteurellosis, it is crucial to avoid animal bites, scratches, and direct contact with animal saliva. A meticulous history-taking, expeditious recognition, and judicious antimicrobial therapy are paramount for the effective management of such infections, with the objective of averting severe complications. In this case, the patient was successfully treated with intravenous antibiotics without the need for surgical intervention, highlighting the importance of a multidisciplinary approach in managing uncommon nosological conditions.
